# 4-[2-(Benzyl­amino)­phen­yl]-2,6-dimethyl­quinoline *N*-oxide

**DOI:** 10.1107/S1600536812011002

**Published:** 2012-03-17

**Authors:** Mario Geffe, Dieter Schollmeyer, Heiner Detert

**Affiliations:** a University Mainz, Duesbergweg 10-14, 55099 Mainz, Germany

## Abstract

The title compound, C_24_H_22_N_2_O, was obtained in a two-step procedure from the corresponding 4-(2-iodo­phen­yl)quinoline. The quinoline system is approximately planar [maximum deviation from the least-squares plane = 0.021 (2) Å]. The planes of the quinoline system and the phenyl ring subtend a dihedral angle of 78.08 (8)°. In the crystal, pairs of mol­ecules are connected *via* a center of symmetry and linked by a pair of angular N—H⋯O hydrogen bond. These dimers form columns oriented along the *c* axis.

## Related literature

For aminations of iodo­lium salts, see: Letessier *et al.* (2011**a* ▶,b* ▶), Letessier & Detert (2012 ▶). For quinoline *N*-oxides, see: Moreno-Fuquen *et al.* (2007 ▶); Ivashevskaja *et al.* (2002 ▶); Fahl­quist *et al.* (2006 ▶). For heteroanalogous carbazoles, see: Dassonneville *et al.* (2010 ▶, 2011 ▶); Nissen & Detert (2011 ▶). For Buchwald-Hartwig amination, see: Hartwig (1999 ▶); Muci & Buchwald (2002 ▶). For twist of *o*-substituted biaryls, see: Miao *et al.* (2009 ▶); Moschel *et al.* (2011 ▶).
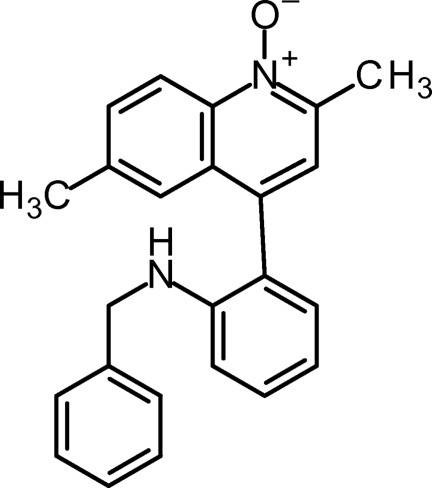


## Experimental

### 

#### Crystal data

C_24_H_22_N_2_O
*M* *_r_* = 354.44
Monoclinic, 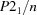

*a* = 10.1656 (3) Å
*b* = 14.1135 (5) Å
*c* = 12.9372 (4) Å
β = 91.547 (3)°
*V* = 1855.46 (11) Å^3^
*Z* = 4
Cu *K*α radiation
μ = 0.61 mm^−1^
*T* = 193 K
0.26 × 0.18 × 0.18 mm

#### Data collection

Stoe IPDS 2T diffractometer
18005 measured reflections
3120 independent reflections
2803 reflections with *I* > 2σ(*I*)
*R*_int_ = 0.034

#### Refinement

*R*[*F* ^2^ > 2σ(*F* ^2^)] = 0.050
*wR*(*F* ^2^) = 0.154
*S* = 1.10
3120 reflections
246 parameters
H-atom parameters constrained
Δρ_max_ = 0.17 e Å^−3^
Δρ_min_ = −0.18 e Å^−3^

### 

Data collection: *X-AREA* (Stoe & Cie, 2011) ▶; cell refinement: *X-AREA* ▶; data reduction: *X-RED* (Stoe & Cie, 2011) ▶; program(s) used to solve structure: *SIR97* (Altomare *et al.*, 1999 ▶); program(s) used to refine structure: *SHELXL97* (Sheldrick, 2008 ▶); molecular graphics: *PLATON* (Spek, 2009 ▶); software used to prepare material for publication: *PLATON*.

## Supplementary Material

Crystal structure: contains datablock(s) I, global. DOI: 10.1107/S1600536812011002/bt5844sup1.cif

Structure factors: contains datablock(s) I. DOI: 10.1107/S1600536812011002/bt5844Isup2.hkl

Supplementary material file. DOI: 10.1107/S1600536812011002/bt5844Isup3.cml

Additional supplementary materials:  crystallographic information; 3D view; checkCIF report

## Figures and Tables

**Table 1:** Hydrogen-bond geometry (Å, °)

*D*—H⋯*A*	*D*—H	H⋯*A*	*D*⋯*A*	*D*—H⋯*A*
N7—H7⋯O24^i^	0.96	2.03	2.7852 (17)	134

Symmetry code: (i) 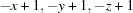.

**Fig. 1.**
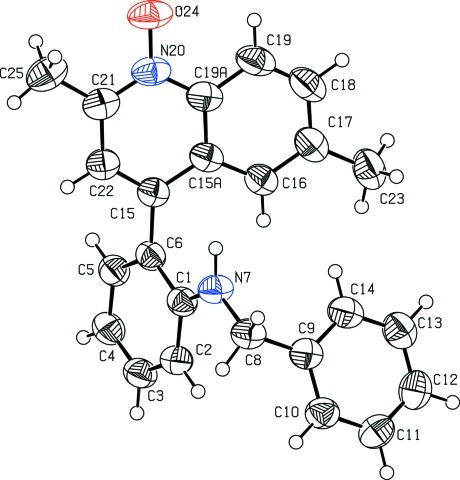
View of compound I. Displacement ellipsoids are drawn at the 50% probability level.

## supplementary crystallographic information

### Comment

As part of a larger project on the synthesis of heteroanalogous carbazoles (Dassonneville *et al.* (2011), Dassonneville *et al.* (2010); Nissen & Detert (2011) iodolium salts became interesting as intermediates. Their twofold Buchwald-Hartwig amination with primary amines results in the formation of the pyrrole ring leading to carbazoles (Letessier *et al.* (2011*a*)) or carbolines (Letessier *et al.* (2011*b*), Letessier & Detert (2012)). The attempted formation of a benzo-quinolino-annulated iodolium salt *via* oxidation of the 4-(2-iodophenyl)quinoline and electrophilic ring closure failed. A mixture of two compounds, probably the iodosophenyl-quinoline and the iodophenyl-quinoline-*N*-oxide was obtained instead. Upon standing in chloroform solution, the former slowly isomerizes to the latter compound. Buchwald-Hartwig amination of the inseparable mixture with benzyl amine and Pd_2_(dba)_3_/Xantphos as catalytic system results in the formation of the title compound (*ca* 35%).

The title compound crystallizes as a centrosymmetric dimer stabilized by hydrogen bonding from the amino group to the N-oxide. The dimers are arranged in independent columns along the *c* axis. The bonds N7—H7 (0.9629 Å) and H7—O24 (2.03 Å) open an angle of 134°.

The quinoline framework is essentially planar with a maximal deviation of 0.021 (2) Å at C17 from the mean square plane. The dihedral angle between the mean planes of the quinoline and the adjacent phenyl ring is 64.61 (6)° and the mean planes of the phenyl rings open an angle of 78.08 (8)°. The amino group is coplanar with the mean plane of the phenyl ring: C8—N7-phenyl: 0.128 (2)°.

### Experimental

2,6-Dimethyl-4-(2-iodophenyl)quinoline (53.9 mg, 0.15 mmol) was dissolved in 1.5 ml of dichloromethane in a flame-dried Schlenk tube and at 273 K. 11.6 µ*L*(17.1 mg, 0.15 mmol) of trifluoromethane sulfonic acid were added. While stirring and cooling, *m*CPBA (38.8 mg, 0.23 mmol) was added. After 10 min trifluoromethansulfonic acid (23.3 µ*L*, 34.2 mg, 0.3 mmol) was added and stirring continued for 30 min. The solvent was removed *in vacuo*, diethyl ether (5 ml) was added. An oily layer separated which crystallized upon standing for 8 h. The crystalline solid was isolated by suction filtration and washed with cold ether. Yield: 27.8 mg of a mixture of two compounds (*ca* 1: 0.6). In a Schlenk tube, this product (380 mg) was suspended in toluene (10 ml) and benzyl amine (96.4 mg, 0.9 mmol), Pd_2_(dba)_3_ (27.6 mg, 0.03 mmol), Xanthphos (52.2 mg, 0.09 mmol) and Cs_2_CO_3_ (684.3 mg, 2.1 mmol) were added. The mixture was stirred over night at 373 K, cooled, filtered through celite and the filter cake was washed with ethyl acetate (50 ml). The pooled organic solutions were washed with water, brine, and dried over MgSO_4_. After removal of the solvents *in vacuo*, the residue was purified by chromatography on Al_2_O_3_ with gradient elution starting with petroleum ether, followed by ethyl acetate and finally methanol. yield: 124.2 mg (0.35 mmol) of the title compound (*R*_f_ = 0.68 SiO_2_, ethyl acetate/methanol = 1/1) as yellow crystals with m. p. = 497 - 499 K. 4-(2-(Benzylamino)phenyl-2,6-dimethylquinoline (60 mg, 0.18 mmol, colorless solid, m. p. = 448 - 450 K) was isolated as a first fraction (*R*_f_ = 0.56 (SiO_2_, petroleum ether/ethyl acetate = 1/1)

### Refinement

All hydrogen atom were located in a difference Fourier map. Nevertheless, they were refined using a riding model with N—H = 0.96 Å, C—H = 0.95 Å (aromatic) or 0.98–0.99 Å (*sp*^3^ C-atom) and with isotropic displacement parameters set at 1.2–1.5 times of the *U*_eq_ of the parent atom.

### Crystal data

**Table:**

C_24_H_22_N_2_O	*F*(000) = 752
*M**_r_* = 354.44	*D*_x_ = 1.269 Mg m^−^^3^
Monoclinic, *P*2_1_/*n*	Melting point: 449 K
Hall symbol: -P 2yn	Cu *K*α radiation, λ = 1.54178 Å
*a* = 10.1656 (3) Å	Cell parameters from 27155 reflections
*b* = 14.1135 (5) Å	θ = 3.1–68.2°
*c* = 12.9372 (4) Å	µ = 0.61 mm^−^^1^
β = 91.547 (3)°	*T* = 193 K
*V* = 1855.46 (11) Å^3^	Needle, yellow
*Z* = 4	0.26 × 0.18 × 0.18 mm

### Data collection

**Table:**

Stoe IPDS 2T diffractometer	2803 reflections with *I* > 2σ(*I*)
Radiation source: Incoatec microSource Cu	*R*_int_ = 0.034
X-ray mirror monochromator	θ_max_ = 66.5°, θ_min_ = 4.6°
Detector resolution: 6.67 pixels mm^-1^	*h* = −12→12
rotation method scans	*k* = −16→16
18005 measured reflections	*l* = −13→13
3120 independent reflections	

### Refinement

**Table:**

Refinement on *F*^2^	Primary atom site location: structure-invariant direct methods
Least-squares matrix: full	Secondary atom site location: difference Fourier map
*R*[*F*^2^ > 2σ(*F*^2^)] = 0.050	Hydrogen site location: inferred from neighbouring sites
*wR*(*F*^2^) = 0.154	H-atom parameters constrained
*S* = 1.10	*w* = 1/[σ^2^(*F*_o_^2^) + (0.099*P*)^2^ + 0.2406*P*] where *P* = (*F*_o_^2^ + 2*F*_c_^2^)/3
3120 reflections	(Δ/σ)_max_ < 0.001
246 parameters	Δρ_max_ = 0.17 e Å^−^^3^
0 restraints	Δρ_min_ = −0.18 e Å^−^^3^

### Special details

**Table:**

Experimental. ^1^H-NMR (400 MHz, CDCl_3_): δ = 8.71 (d, ^3^J = 8.9 Hz, 1 H), 7.59 (dd, ^3^J = 8.9 Hz, 4 J = 1.7 Hz, 1 H), 7.43 (s, 1 H), 7.36 - 7.16 (m, 7 H), 7.11 (dd, 3 J = 7.4 Hz, ^4^J = 1.5 Hz, 1 H), 6.84 (t, ^3^J = 7.4 Hz, 1 H), 6.75 (d, ^3^J = 8.2 Hz, 1 H), 4.30 (s, 2 H, CH_2_), 2.71 (s, 3 H, CH_3_), 2.47 (s, 3 H, CH_3_).^13^C-NMR (75 MHz, CDCl_3_): δ = 145.6, 144.0, 140.4, 139.7, 137.0, 133.4, 131.8, 130.3, 129.4, 128.3 (2 C), 128.0, 126.7 (2 C), 126.5, 125.5, 125.1, 121.8, 119.4, 115.7, 110.4, 45.9, 21.0, 18.2.IR (ATR) ν = 3324, 3016, 2910, 2857, 1597, 1573, 1520, 1410, 1385, 1319, 1300, 1237, 1201, 1162, 1105, 982, 925, 872, 823, 795, 738, 699 cm^-1^.ESI-MS: 355.2 (*M*+H)^+^
Geometry. All e.s.d.'s (except the e.s.d. in the dihedral angle between two l.s. planes) are estimated using the full covariance matrix. The cell e.s.d.'s are taken into account individually in the estimation of e.s.d.'s in distances, angles and torsion angles; correlations between e.s.d.'s in cell parameters are only used when they are defined by crystal symmetry. An approximate (isotropic) treatment of cell e.s.d.'s is used for estimating e.s.d.'s involving l.s. planes.
Refinement. Refinement of *F*^2^ against ALL reflections. The weighted *R*-factor *wR* and goodness of fit *S* are based on *F*^2^, conventional *R*-factors *R* are based on *F*, with *F* set to zero for negative *F*^2^. The threshold expression of *F*^2^ > σ(*F*^2^) is used only for calculating *R*-factors(gt) *etc*. and is not relevant to the choice of reflections for refinement. *R*-factors based on *F*^2^ are statistically about twice as large as those based on *F*, and *R*- factors based on ALL data will be even larger.

### Fractional atomic coordinates and isotropic or equivalent isotropic displacement parameters (Å^2^)

**Table:**

	*x*	*y*	*z*	*U*_iso_*/*U*_eq_	
C1	0.71651 (14)	0.61537 (10)	0.20685 (11)	0.0467 (4)	
C2	0.77995 (15)	0.65594 (11)	0.12222 (12)	0.0519 (4)	
H2	0.7410	0.7089	0.0880	0.062*	
C3	0.89744 (16)	0.62050 (12)	0.08793 (13)	0.0561 (4)	
H3	0.9384	0.6496	0.0309	0.067*	
C4	0.95605 (15)	0.54320 (12)	0.13561 (13)	0.0570 (4)	
H4	1.0372	0.5190	0.1122	0.068*	
C5	0.89426 (14)	0.50159 (11)	0.21827 (13)	0.0520 (4)	
H5	0.9344	0.4485	0.2513	0.062*	
C6	0.77536 (14)	0.53505 (10)	0.25443 (11)	0.0461 (4)	
N7	0.60350 (13)	0.65335 (9)	0.24471 (10)	0.0533 (4)	
H7	0.5685	0.6237	0.3051	0.064*	
C8	0.53902 (15)	0.73596 (11)	0.20054 (12)	0.0533 (4)	
H8A	0.6068	0.7844	0.1866	0.064*	
H8B	0.4795	0.7627	0.2524	0.064*	
C9	0.46014 (14)	0.71777 (10)	0.10157 (12)	0.0507 (4)	
C10	0.46398 (16)	0.78089 (11)	0.01988 (13)	0.0572 (4)	
H10	0.5201	0.8346	0.0249	0.069*	
C11	0.38731 (18)	0.76708 (12)	−0.06926 (14)	0.0637 (5)	
H11	0.3914	0.8110	−0.1247	0.076*	
C12	0.30522 (17)	0.68959 (13)	−0.07725 (15)	0.0640 (5)	
H12	0.2516	0.6805	−0.1377	0.077*	
C13	0.30119 (17)	0.62530 (13)	0.00294 (16)	0.0660 (5)	
H13	0.2456	0.5713	−0.0028	0.079*	
C14	0.37786 (16)	0.63914 (12)	0.09166 (14)	0.0608 (4)	
H14	0.3744	0.5945	0.1465	0.073*	
C15	0.71631 (14)	0.48715 (10)	0.34537 (12)	0.0474 (4)	
C15A	0.59375 (14)	0.43752 (10)	0.33858 (12)	0.0491 (4)	
C16	0.51672 (14)	0.43056 (10)	0.24628 (13)	0.0520 (4)	
H16	0.5461	0.4613	0.1858	0.062*	
C17	0.40017 (15)	0.38053 (11)	0.24148 (15)	0.0596 (4)	
C18	0.35773 (17)	0.33600 (13)	0.33235 (18)	0.0695 (5)	
H18	0.2770	0.3020	0.3302	0.083*	
C19	0.42868 (17)	0.34010 (12)	0.42324 (17)	0.0670 (5)	
H19	0.3978	0.3092	0.4832	0.080*	
C19A	0.54775 (15)	0.39039 (11)	0.42727 (13)	0.0549 (4)	
N20	0.62225 (14)	0.39157 (10)	0.51958 (11)	0.0584 (4)	
C21	0.73692 (17)	0.43875 (11)	0.52665 (13)	0.0567 (4)	
C22	0.78247 (16)	0.48609 (11)	0.43914 (12)	0.0529 (4)	
H22	0.8636	0.5192	0.4452	0.063*	
C23	0.31976 (18)	0.37395 (14)	0.14289 (18)	0.0732 (5)	
H23A	0.2438	0.4165	0.1465	0.110*	
H23B	0.2890	0.3087	0.1330	0.110*	
H23C	0.3739	0.3924	0.0846	0.110*	
O24	0.57999 (13)	0.34483 (10)	0.59927 (10)	0.0755 (4)	
C25	0.8079 (2)	0.43775 (15)	0.62843 (14)	0.0725 (5)	
H25A	0.8872	0.4770	0.6248	0.109*	
H25B	0.8328	0.3726	0.6462	0.109*	
H25C	0.7505	0.4631	0.6815	0.109*	

### Atomic displacement parameters (Å^2^)

**Table:**

	*U*^11^	*U*^22^	*U*^33^	*U*^12^	*U*^13^	*U*^23^
C1	0.0483 (8)	0.0467 (8)	0.0453 (8)	−0.0049 (6)	0.0027 (6)	−0.0022 (6)
C2	0.0552 (8)	0.0504 (8)	0.0504 (9)	−0.0038 (6)	0.0054 (6)	0.0039 (6)
C3	0.0553 (9)	0.0606 (9)	0.0529 (9)	−0.0096 (7)	0.0093 (7)	0.0038 (7)
C4	0.0469 (8)	0.0643 (10)	0.0603 (10)	−0.0025 (7)	0.0106 (7)	0.0007 (7)
C5	0.0482 (8)	0.0514 (8)	0.0565 (9)	−0.0014 (6)	0.0029 (7)	0.0016 (6)
C6	0.0469 (7)	0.0450 (7)	0.0464 (8)	−0.0060 (6)	0.0025 (6)	−0.0020 (6)
N7	0.0584 (8)	0.0509 (7)	0.0513 (8)	0.0061 (5)	0.0123 (6)	0.0076 (5)
C8	0.0588 (9)	0.0458 (8)	0.0557 (9)	0.0033 (6)	0.0101 (7)	0.0010 (6)
C9	0.0492 (8)	0.0465 (8)	0.0569 (9)	0.0076 (6)	0.0108 (6)	0.0007 (6)
C10	0.0603 (9)	0.0472 (8)	0.0641 (10)	0.0020 (7)	0.0032 (7)	0.0051 (7)
C11	0.0701 (10)	0.0568 (9)	0.0638 (11)	0.0095 (8)	−0.0024 (8)	0.0065 (8)
C12	0.0578 (9)	0.0650 (10)	0.0688 (11)	0.0128 (8)	−0.0036 (8)	−0.0081 (8)
C13	0.0590 (10)	0.0599 (10)	0.0792 (12)	−0.0035 (7)	0.0018 (8)	−0.0045 (8)
C14	0.0603 (9)	0.0541 (9)	0.0683 (11)	−0.0014 (7)	0.0103 (8)	0.0060 (8)
C15	0.0491 (8)	0.0420 (7)	0.0513 (9)	0.0026 (6)	0.0068 (6)	0.0002 (6)
C15A	0.0494 (8)	0.0400 (7)	0.0584 (9)	0.0041 (6)	0.0122 (6)	0.0014 (6)
C16	0.0507 (8)	0.0435 (8)	0.0621 (10)	0.0006 (6)	0.0084 (7)	−0.0015 (6)
C17	0.0486 (8)	0.0467 (8)	0.0838 (12)	0.0007 (6)	0.0078 (8)	−0.0028 (7)
C18	0.0492 (9)	0.0546 (9)	0.1052 (16)	−0.0036 (7)	0.0162 (9)	0.0077 (9)
C19	0.0559 (9)	0.0568 (10)	0.0895 (13)	0.0044 (7)	0.0254 (9)	0.0170 (8)
C19A	0.0535 (8)	0.0469 (8)	0.0651 (11)	0.0093 (6)	0.0174 (7)	0.0074 (7)
N20	0.0649 (8)	0.0536 (8)	0.0578 (9)	0.0140 (6)	0.0227 (6)	0.0111 (6)
C21	0.0651 (9)	0.0525 (8)	0.0530 (10)	0.0118 (7)	0.0120 (7)	0.0027 (7)
C22	0.0574 (8)	0.0491 (8)	0.0523 (9)	0.0026 (6)	0.0052 (7)	0.0007 (6)
C23	0.0560 (10)	0.0622 (10)	0.1011 (15)	−0.0063 (8)	−0.0062 (9)	−0.0083 (9)
O24	0.0814 (8)	0.0774 (9)	0.0694 (9)	0.0177 (6)	0.0337 (7)	0.0280 (6)
C25	0.0907 (13)	0.0757 (12)	0.0512 (10)	0.0172 (10)	0.0075 (9)	0.0057 (8)

### Geometric parameters (Å, º)

**Table:**

C1—N7	1.370 (2)	C13—H13	0.9500
C1—C2	1.407 (2)	C14—H14	0.9500
C1—C6	1.415 (2)	C15—C22	1.371 (2)
C2—C3	1.379 (2)	C15—C15A	1.430 (2)
C2—H2	0.9500	C15A—C16	1.414 (2)
C3—C4	1.381 (2)	C15A—C19A	1.417 (2)
C3—H3	0.9500	C16—C17	1.379 (2)
C4—C5	1.385 (2)	C16—H16	0.9500
C4—H4	0.9500	C17—C18	1.411 (3)
C5—C6	1.390 (2)	C17—C23	1.499 (3)
C5—H5	0.9500	C18—C19	1.364 (3)
C6—C15	1.497 (2)	C18—H18	0.9500
N7—C8	1.448 (2)	C19—C19A	1.403 (2)
N7—H7	0.9629	C19—H19	0.9500
C8—C9	1.514 (2)	C19A—N20	1.397 (2)
C8—H8A	0.9900	N20—O24	1.3064 (17)
C8—H8B	0.9900	N20—C21	1.343 (2)
C9—C10	1.384 (2)	C21—C22	1.404 (2)
C9—C14	1.393 (2)	C21—C25	1.484 (3)
C10—C11	1.388 (2)	C22—H22	0.9500
C10—H10	0.9500	C23—H23A	0.9800
C11—C12	1.378 (3)	C23—H23B	0.9800
C11—H11	0.9500	C23—H23C	0.9800
C12—C13	1.380 (3)	C25—H25A	0.9800
C12—H12	0.9500	C25—H25B	0.9800
C13—C14	1.384 (3)	C25—H25C	0.9800
			
N7—C1—C2	121.68 (14)	C9—C14—H14	119.6
N7—C1—C6	120.44 (13)	C22—C15—C15A	117.05 (14)
C2—C1—C6	117.85 (13)	C22—C15—C6	120.19 (13)
C3—C2—C1	121.45 (15)	C15A—C15—C6	122.70 (14)
C3—C2—H2	119.3	C16—C15A—C19A	117.66 (14)
C1—C2—H2	119.3	C16—C15A—C15	123.22 (14)
C2—C3—C4	120.66 (15)	C19A—C15A—C15	119.10 (15)
C2—C3—H3	119.7	C17—C16—C15A	121.95 (15)
C4—C3—H3	119.7	C17—C16—H16	119.0
C3—C4—C5	118.73 (15)	C15A—C16—H16	119.0
C3—C4—H4	120.6	C16—C17—C18	118.18 (17)
C5—C4—H4	120.6	C16—C17—C23	121.20 (16)
C4—C5—C6	122.15 (15)	C18—C17—C23	120.62 (15)
C4—C5—H5	118.9	C19—C18—C17	122.21 (16)
C6—C5—H5	118.9	C19—C18—H18	118.9
C5—C6—C1	119.14 (13)	C17—C18—H18	118.9
C5—C6—C15	118.80 (13)	C18—C19—C19A	119.28 (17)
C1—C6—C15	122.01 (13)	C18—C19—H19	120.4
C1—N7—C8	123.31 (13)	C19A—C19—H19	120.4
C1—N7—H7	116.8	N20—C19A—C19	119.02 (15)
C8—N7—H7	119.8	N20—C19A—C15A	120.24 (14)
N7—C8—C9	114.90 (13)	C19—C19A—C15A	120.72 (17)
N7—C8—H8A	108.5	O24—N20—C21	119.97 (15)
C9—C8—H8A	108.5	O24—N20—C19A	119.11 (14)
N7—C8—H8B	108.5	C21—N20—C19A	120.91 (13)
C9—C8—H8B	108.5	N20—C21—C22	119.03 (15)
H8A—C8—H8B	107.5	N20—C21—C25	117.11 (15)
C10—C9—C14	118.15 (15)	C22—C21—C25	123.85 (16)
C10—C9—C8	120.76 (14)	C15—C22—C21	123.65 (15)
C14—C9—C8	121.04 (14)	C15—C22—H22	118.2
C9—C10—C11	121.16 (16)	C21—C22—H22	118.2
C9—C10—H10	119.4	C17—C23—H23A	109.5
C11—C10—H10	119.4	C17—C23—H23B	109.5
C12—C11—C10	119.94 (16)	H23A—C23—H23B	109.5
C12—C11—H11	120.0	C17—C23—H23C	109.5
C10—C11—H11	120.0	H23A—C23—H23C	109.5
C11—C12—C13	119.73 (16)	H23B—C23—H23C	109.5
C11—C12—H12	120.1	C21—C25—H25A	109.5
C13—C12—H12	120.1	C21—C25—H25B	109.5
C12—C13—C14	120.23 (16)	H25A—C25—H25B	109.5
C12—C13—H13	119.9	C21—C25—H25C	109.5
C14—C13—H13	119.9	H25A—C25—H25C	109.5
C13—C14—C9	120.79 (16)	H25B—C25—H25C	109.5
C13—C14—H14	119.6		
			
N7—C1—C2—C3	176.45 (14)	C6—C15—C15A—C16	−1.0 (2)
C6—C1—C2—C3	−1.6 (2)	C22—C15—C15A—C19A	−0.2 (2)
C1—C2—C3—C4	0.5 (2)	C6—C15—C15A—C19A	177.09 (13)
C2—C3—C4—C5	0.3 (2)	C19A—C15A—C16—C17	0.5 (2)
C3—C4—C5—C6	0.1 (2)	C15—C15A—C16—C17	178.62 (14)
C4—C5—C6—C1	−1.3 (2)	C15A—C16—C17—C18	0.4 (2)
C4—C5—C6—C15	−178.91 (14)	C15A—C16—C17—C23	179.98 (14)
N7—C1—C6—C5	−176.09 (14)	C16—C17—C18—C19	−0.8 (3)
C2—C1—C6—C5	2.0 (2)	C23—C17—C18—C19	179.66 (16)
N7—C1—C6—C15	1.4 (2)	C17—C18—C19—C19A	0.2 (3)
C2—C1—C6—C15	179.53 (13)	C18—C19—C19A—N20	−177.79 (14)
C2—C1—N7—C8	1.3 (2)	C18—C19—C19A—C15A	0.7 (2)
C6—C1—N7—C8	179.29 (13)	C16—C15A—C19A—N20	177.44 (12)
C1—N7—C8—C9	77.05 (18)	C15—C15A—C19A—N20	−0.8 (2)
N7—C8—C9—C10	−138.41 (15)	C16—C15A—C19A—C19	−1.1 (2)
N7—C8—C9—C14	44.11 (19)	C15—C15A—C19A—C19	−179.29 (14)
C14—C9—C10—C11	0.6 (2)	C19—C19A—N20—O24	0.9 (2)
C8—C9—C10—C11	−176.96 (14)	C15A—C19A—N20—O24	−177.63 (13)
C9—C10—C11—C12	0.2 (2)	C19—C19A—N20—C21	179.90 (15)
C10—C11—C12—C13	−1.0 (3)	C15A—C19A—N20—C21	1.4 (2)
C11—C12—C13—C14	0.9 (3)	O24—N20—C21—C22	178.06 (13)
C12—C13—C14—C9	−0.0 (3)	C19A—N20—C21—C22	−0.9 (2)
C10—C9—C14—C13	−0.7 (2)	O24—N20—C21—C25	−2.3 (2)
C8—C9—C14—C13	176.86 (15)	C19A—N20—C21—C25	178.73 (13)
C5—C6—C15—C22	61.76 (19)	C15A—C15—C22—C21	0.6 (2)
C1—C6—C15—C22	−115.76 (16)	C6—C15—C22—C21	−176.72 (14)
C5—C6—C15—C15A	−115.43 (16)	N20—C21—C22—C15	−0.1 (2)
C1—C6—C15—C15A	67.05 (19)	C25—C21—C22—C15	−179.72 (15)
C22—C15—C15A—C16	−178.31 (13)		

### Hydrogen-bond geometry (Å, º)

**Table:**

*D*—H···*A*	*D*—H	H···*A*	*D*···*A*	*D*—H···*A*
N7—H7···O24^i^	0.96	2.03	2.7852 (17)	134

Symmetry code: (i) −*x*+1, −*y*+1, −*z*+1.
